# ATTITUDES AND PRACTICE PATTERNS OF CANADIAN PHYSIATRISTS REGARDING MEDICAL CANNABIS

**DOI:** 10.2340/jrm-cc.v8.43254

**Published:** 2025-12-03

**Authors:** Karen Ethans, Harpal Chaudhary, Alan Casey, Colleen O’Connell, Mayur Nankar, Avni Khandelwal

**Affiliations:** 1 Section of Physical Medicine and Rehabilitation, Department of Internal Medicine, Rady Faculty of Health Sciences, University of Manitoba, Winnipeg, Canada; 2 Department of Rehabilitation, Stan Cassidy Centre for Rehabilitation, Horizon Health Network, Fredericton, Canada; 3 Department of Physical Medicine and Rehabilitation, Health Sciences Centre, Winnipeg, Canada

**Keywords:** medical cannabis, physiatry, neuropathic pain

## Abstract

**Objective:**

To assess practice patterns and attitudes of Canadian physiatrists, given their expertise in pain management and spasticity, conditions in which medical cannabis (MC) should be considered.

**Design:**

A 24-item, survey questionnaire was sent to physiatrists across Canada.

**Subjects:**

One hundred and nine physiatrists responded.

**Methods:**

A structured web-based survey distributed to members of Canadian Association of Physical Medicine and Rehabilitation. Inferential statistical analysis was conducted.

**Results:**

A majority of respondents acknowledged the medicinal value of MC, with 61% of respondents feeling comfortable discussing it, whereas only 31% felt comfortable authorizing MC. Years of work experience did not impact comfort regarding discussions of MC, but those with 21+ years of experience authorized MC more frequently. A significant relationship was observed between subspecialty and MC prescribing; most prescriptions authorized for neuropathic pain, musculoskeletal pain and spasticity. Most respondents agreed that medical school and residency programs provided insufficient education on MC, and that governmental and institutional guidelines remained unclear.

**Conclusion:**

Addressing cannabinoids in medical school and residency is important to improve the therapeutic and counselling aspects of patient care in addressing safety and preventing misuse. With clearer guidelines and more research on MC efficacy, physiatrists will be more knowledgeable and better able to improve patient lives.

## LAY ABSTRACT

Canadian physiatrists show expertise in conditions in which medical cannabis (MC) should be considered, showcasing the importance of capturing this demographics attitudes and practice patterns on MC. A 24-item, web-based survey was sent to physiatrists across Canada. The results show a large consensus for agreement in the medicinal value of MC and comfort discussing it with patients, but lack of confidence in authorization patterns. Respondents agreed that their medical school and residency training left them with considerable gaps in MC knowledge; and this coupled with the lack of institutional and governmental guidelines deter them from using MC. Though years of clinical experience did not impact doctor-patient discussions of MC, those with 21+ years of experience authorized MC more frequently, perhaps due to first-hand observations of MC benefits for patients.

Medical cannabis (MC) is a highly disputed treatment option amongst medical practitioners and the public, despite being legalized for medical use since 2001. Enthusiasm and cannabis advocacy led to the legalization of cannabis for non-medical use in 2018. However, research is sparse regarding the safety and efficacy of the use of MC, which continues to intensify the mixed views for MC amongst medical practitioners.

Medical literature supports the analgesic effects of MC for neuropathic pain (1–2), non-neuropathic pain (3–6), spasticity (6–8) and fibromyalgia (9–10), although some studies show aggravation in conditions relating to heart failure, arrhythmias and higher blood pressure (11, 12); furthermore, there is a lack of consensus concerning the adverse neuropsychiatry and psychotic disorders (13–16).

There have been several studies conducted worldwide to learn about attitudes of medical practitioners towards MC (17–23). All these studies found that physicians were concerned about the lack of research and knowledge regarding the use of MC. A Canadian survey in 2015, which included general practitioners and specialists, found a large gap between the knowledge currently available and the desired knowledge; practitioners wanted more information on risk and benefit, development of treatment plans and information comparing cannabinoid products (1).

There appeared to be a dispute in opinion amongst physicians regarding the use of MC as a “medication.” Advocates of MC believed that the use was justified as an effective means to improve the quality of life of their patients (24), whereas others did not feel comfortable due to the lack of rigorous studies (25). Even presently, there is a shortage of large volume research studies to support MC literature. Physicians were concerned about the insufficient training received during their medical school or respective residencies (17–18, 20–23). Furthermore, the unclear current legislation and guidelines were frequently mentioned concerns by many practitioners (18, 20).

Physiatrists specialize in symptom management of people with chronic neurologic and musculoskeletal conditions, including neuropathic pain, spasticity, and soft tissue, bone and joint pain. As this specialty group has not been specifically studied, we believe in the importance in engaging in an individual study analysing physiatrist practice patterns and attitudes towards MC.

## METHODS

A 24-item questionnaire was developed and distributed to practicing Canadian physiatrists in 2015 to assess their attitudes and practice patterns regarding MC. This study was reviewed and approved by the Research Ethics Board at the University of Manitoba, Winnipeg, Manitoba.

Surveys were conducted anonymously through a web-based platform, Survey Monkey^TM^, and was sent to the members of the Canadian Association of Physical Medicine and Rehabilitation (CAPMR) (*n* = 348). The survey contained a variety of categorical/nominal data, ordinal data and ratio/interval data.

*Statistical analysis*. Analysis was conducted using SPSS v 22 IBM, Chicago, Illinois. Chi-square analyses were used to test hypotheses for categorical data. Significant interactions were followed by post hoc Bonferroni corrections. Standardized residuals were calculated from observed and expected frequencies and then were compared to *z*-scores 1.96 (α < 0.05) and 2.56 (α < 0.01) based on factor levels to determine statistical significance. Statistical significance was set at α < 0.05. The variables tested included:

- Authorization of MC dependent on practice settings.
- Effect of practice setting on comfort level between physiatrist and patient on MC.
- Practice patterns based on years practiced.
- Comfort level based on years practiced.
- Frequency of MC authorization by subspecialty.

*Demographics*. Out of 348, 109 physiatrists responded (31%). In this 24-item survey, a few respondents chose to omit some questions; however, this percentage was negligible for most questions and was accounted for in the analysis when appropriate. Overall, the survey respondents represented a diverse range of practice settings and levels of experience (Table I).

**Table I:** Demographics – practice settings, subspecialty and number of years practiced

Practice settings	Responses	*N* (108)
Academic centre	55.56%	60
Private practice	24.07%	26
Tertiary care hospital	9.26%	10
Others	11.11%	12
*Subspeciality*		*N* (105)
Neurological rehabilitation	22.86%	24
General physiatry	22.86%	24
Musculoskeletal rehabilitation	11.43%	12
Spinal cord injury rehabilitation	9.52%	10
Pain medicine	9.52%	10
Amputee rehabilitation	6.67%	7
Electro-diagnostics	4.76%	5
Medico-legal	1.90%	2
Other	10.48%	11
*No. of years practiced*		*N* (105)
Less than 5 years	26.67%	28
6–10 years	20.00%	21
11–20 years	25.71%	27
21–30 years	15.24%	16
More than 30 years	12.38%	13

*N* = sample size.

## RESULTS

*Perceived therapeutic value of MC*. Most respondents (*n* = 91, 83%) agreed that cannabis has medicinal value. Seventy-five per cent agreed that ingestion of cannabis in a vaporized or oral form, rather than smoking, had medicinal value. Sixty-three per cent agreed that cannabis efficacy varied depending on the content of tetrahydrocannabinol (THC) and cannabidiol (CBD). Furthermore, 68% respondents agreed that THC-related side effects differ significantly from those of CBD.

*Physiatrists’ comfort levels with the use of MC*. Respondents were asked about their comfort discussing MC with their patients. The majority (61%, *n* = 66) agreed with feeling comfortable discussing MC; however, approximately half (51%) did not feel comfortable signing documents to authorize the use of MC. Physiatrists who had been practicing for less than 20 years accounted for most of those reporting discomfort.

*Authorization of medical document, frequency and requests for authorization*. Interestingly, forty-two per cent of respondents reported never authorizing MC. Subspecialties who were most likely to authorize MC most included neurological rehabilitation, general physiatry, spinal cord injury (SCI) rehabilitation and pain medicine. Amongst those who responded regarding prerequisites for prescribing MC, 67% responded; of these, 67 participants reported trialling other medications first, whilst 35 respondents prescribed other cannabinoid products with THC ± CBD products (eg. nabilone and nabiximol) prior to prescribing MC. Many (44%) stated that 0–5% of their patients requested MC, whilst only 11% reported requests from > 20% of their patients. Individuals diagnosed with neuropathic pain, chronic musculoskeletal pain, spasticity and fibromyalgia accounted for the highest proportion of patients given MC (Table II). Additionally, 61% of respondents agreed that some patient requests were for recreational purposes. Table III summarizes factors influencing likelihood for that physician to authorization MC.

**Table II:** Frequency of MC authorization by medical condition

Conditions	%	*N* (102)
Neuropathic pain	76.47	78
Chronic musculoskeletal pain	67.65	69
Spasticity	44.12	45
Fibromyalgia	30.39	31
Anxiety	21.57	22
PTSD	9.80	10
Others	16.67	17

Respondents could indicate more than 1. MC = medical cannabis; PTSD = post-traumatic stress disorder.

**Table III:** Factors influencing likelihood of authorization patterns of MC by physiatrists

Factors	Stronglyagree	Somewhatagree	Neutral/Noresponse	Somewhatdisagree	Stronglydisagree
Current research	3.85%	29.81%	14.42%	30.77%	21.15%
Health Canada guidelines	0.97%	19.42%	29.13%	35.92%	14.56%
CMA, CMPA and provincial guidelines	1.94%	24.27%	35.92%	29.13%	8.74%
Medical school training	1.92%	13.46%	8.65%	23.08%	52.88%
Access to CPD programs on MC	9.71%	32.04%	25.24%	23.30%	9.71%
Clinical guidelines	39.05%	43.81%	9.52%	3.81%	3.81%
Cannabis legalization	2.88%	29.81%	24.04%	20.19%	23.08%

MC: medical cannabis; CMA: Canadian Medical Association; CMPA: Canadian Medical Protective Association; CPD: Continuing Professional Development.

*Perception of education and guidelines regarding the use of MC and authorization*. Regarding the adequacy of research, 49% disagreed that existing research satisfactorily supports the use of MC, whilst 32% agreed. Almost half (48%) disagreed that Health Canada guidelines were clear and easy to understand. When asked about the comprehensibility of guidelines from Canadian Medical Association, Canadian Medical Protective Association and provincial colleges, 36% disagreed, 25% agreed and 18% chose no response. Eighty per cent (*n* = 87) of respondents agreed that they would feel more comfortable discussing MC if comprehensible clinical guidelines were implemented.

*Legalization of cannabis*. When asked if legalization for recreational purposes would improve comfort discussing MC, only three respondents strongly agreed. Forty-one per cent (*n* = 45) disagreed that legalization would improve their comfort level.

**Fig. 1:**
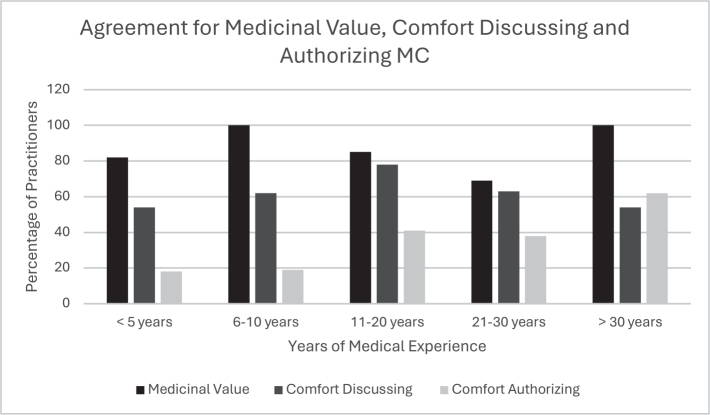
Agreement for medicinal value, and comfort discussing and authorizing MC categorized by years of practice. MC = medical cannabis.

**Fig. 2:**
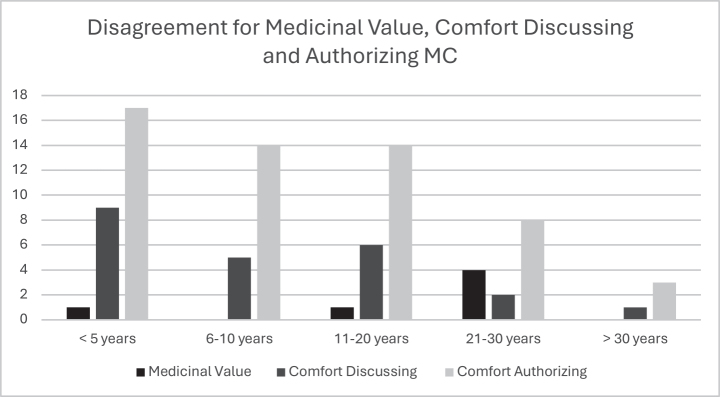
Disagreement on medicinal value, and comfort discussing and authorizing MC categorized by years of practice. MC = medical cannabis.

*Years in practice*. The results on comfort levels by years of practice are shown in Figs. 1 and 2. Overall, the majority agreed that cannabis has a medicinal value, regardless of years of work experience (Fig. 1). Although most practitioners were comfortable discussing MC with their patients, discomfort decreased with increasing years in practice; those practicing < 5 years reported feeling less comfortable (Fig. 2). Physiatrists with more experience were significantly more likely to authorize MC (*p* < 0.001) (Fig. 1).

**Fig. 3:**
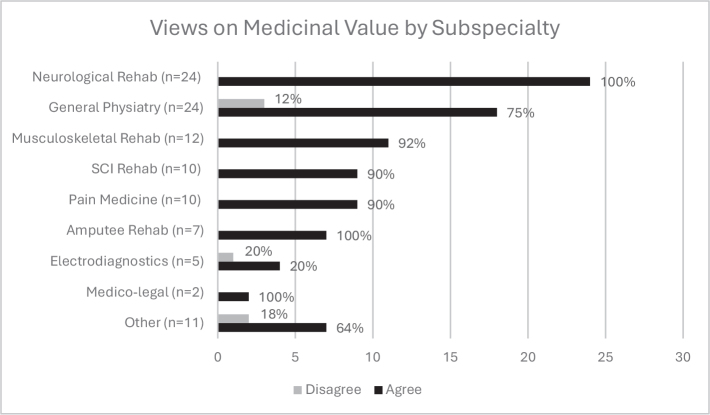
Opinion on the medicinal value of MC by subspecialty. MC = medical cannabis.

**Fig. 4:**
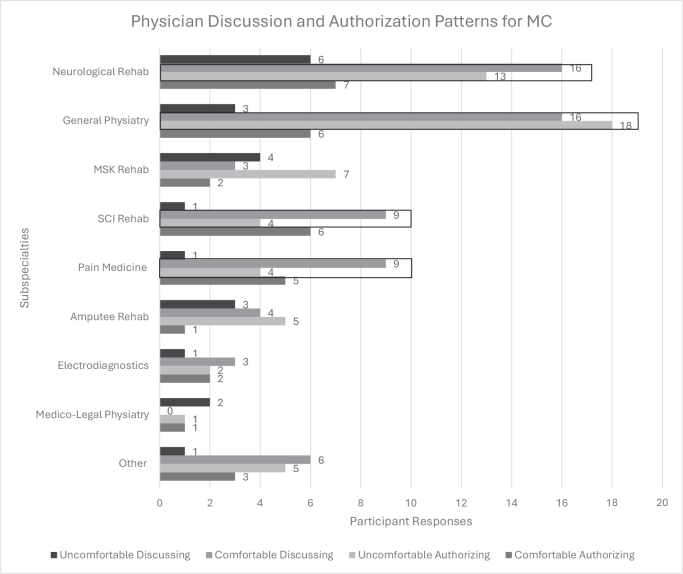
Comfort with discussion and authorization practices for MC by subspecialty. *N* = sample; MC = medical cannabis. This data reflect the absolute number of respondents within each subspecialty who expressed specific opinions. Whilst the number of participants across subspecialties is varied, this disparity is accounted for in this figure. Data highlighted in black boxes showcase an especially wide gap between comfort discussing with patients but discomfort authorizing. Subspecialties include neurological rehabilitation (*n* = 24), general physiatry (*n* = 24), musculoskeletal rehabilitation (*n* = 12), spinal cord injury rehabilitation (*n* = 10), amputee rehabilitation (*n* = 7), electro-diagnostics (*n* = 5), pain medicine (*n* = 10), medico-legal physiatry (*n* = 2) and other (*n* = 11).

*Subspecialty impact*. Across subspecialties, most agreed that cannabis has a medicinal value (Fig. 3) and felt comfortable discussing the use of MC. Figure 4 shows the comfort levels of discussing and authorizing the use of MC across subspecialties, highlighting the discrepancy between comfort in communication but discomfort in utilizing MC as a potential treatment option. A significant interaction was found between subspecialty and frequency of authorizing MC (*p* = 0.030). Physiatrists in SCI rehabilitation authorized MC more than once per week (*p* < 0.01), whilst pain medicine showed a trend towards significance for 5–10 authorizations per year.

*Medical education*. In exploring the area of medical education on MC, 83% of respondents disagreed to receiving adequate training in medical school and 72% disagreed that regarding adequate training during their physical medicine and rehabilitation residency. For access to professional development, 39% agreed to the opportunities related to MC, whilst 31% disagreed.

These data reflect the absolute number of respondents within each subspecialty who expressed specific opinions. Whilst the number of participants across subspecialties is varied, this disparity is accounted for in this figure. Data highlighted in black boxes showcase an especially wide gap between comfort discussing with patients but discomfort authorizing. Subspecialties include neurological rehabilitation (*n* = 24), general physiatry (*n* = 24), musculoskeletal rehabilitation (*n* = 12), SCI rehabilitation (*n* = 10), amputee rehabilitation (*n* = 7), electro-diagnostics (*n* = 5), pain medicine (*n* = 10), medico-legal physiatry (*n* = 2) and other (*n* = 11).

## DISCUSSION

The study objective was to explore attitudes and practice patterns of Canadian physiatrists regarding the use of MC. To our knowledge, this is the first study to examine this sub-population of practitioners. MC is often considered a favourable treatment option for patients suffering from chronic pain, neuropathic pain, spasticity and post-traumatic stress disorder (PTSD).

Results indicated that newer physiatrists were as likely to acknowledge the medicinal benefits of MC and to feel comfortable discussing it with patients though they were less comfortable authorizing its use compared to those with over 11 years of experience. This may be attributed to more first-hand experiences witnessing the therapeutic benefits of MC.

Interestingly, whilst more than half of the participants agreed to feeling comfortable discussing MC, many participants did not feel comfortable authorizing it. Most participants reported inadequate training in both medical school and residency related to MC. This notable discrepancy between respondent willingness to discuss the use of MC but unwillingness to authorize MC usage highlights the tension in treatment implementation, which may reflect the uncertainties regarding evidence, guidelines and insufficient training in MC prescribing (17).

Many respondents believed that research did not provide satisfactory evidence to support the use of MC. A study conducted by Ng et al. noted similar results, and participants had concerns about the long-term effects and lack of rigorous and high-quality studies monitoring vulnerable populations. Additionally, due to the lack of evidence in research, physicians felt that clinical trials have been poorly designed (26). A likely reason for the lack of such high quality, large multi-centred studies is the extreme cost associated with such a study; normally, studies that meet these quality guidelines are patented, pharmaceutical backed medications. However, in Canada, THC and CBD products are not patented, and there are no large industries backing these products, which prevents the typical multi-million-dollar studies that physicians want to see. Some researchers have performed survey and cohort-type studies, including surveys of experienced physicians treating patients with cannabis in their daily practice, and those studies, along with the present one, allow for consideration of the accumulated clinical experience as a source of knowledge and information in guiding physiatrists’ practices.

Previous literature has shown mixed attitudes for the use of MC. This study along with studies conducted in New York (19), Israel (20) and Australia (21) shows physicians generally have positive attitudes towards MC, whilst physicians in Colorado (18) and Ireland (22) showed generally negative overall attitudes. The vast majority of respondents agreed cannabis has a medicinal value (Fig. 1), but physiatrists with less experience felt less comfort discussing it and even more uncomfortable authorizing it, compared to the physiatrists with more experience (Fig. 2).

It was discovered that physiatrists believe that MC has a higher value when utilized in a non-smoked (oral or vaporized) form. Azcarate et al. conducted a study on the use of MC regarding medical conditions and the form of use. Their results supported this belief as smoking form was believed to make recreational use more likely (27). Additionally, it is generally believed that smoked/combusted forms are more hazardous to health than when vaporized or used orally. Also, the smoked form allows for rapid onset with short lasting effects, making it an ineffective medication for patients with a chronic, constant condition such as pain and spasticity that need longer acting, stable dose in an oral form.

The subspecialties that prescribed MC most often included neurological rehabilitation, SCI rehabilitation, pain medicine and general physiatry. Previous studies serve as evidence in supporting the use of cannabinoids as treatment for pain and spinal medicine (28–29); hence, explaining why physiatrists in these specialties are more likely to be treating patients with MC.

Some respondents chose “neutral” when answering whether there are different side effect profiles of cannabis with predominant THC as opposed to CBD. This could be due to (*i*) an absence in comparison trials between THC vs CBD, as only anecdotal evidence currently exists, and most research has been done using THC dominant strains only, and/or (*ii*) participants remaining neutral may not have been familiar with cannabinoids to express an opinion at the time.

Although this study was conducted prior to legalization of cannabis, study results are important as it is still mandatory to have a physician authorize the use of MC in Canada, and the patient cannot access MC without this authorization. There are many benefits of accessing a medical source as opposed to a recreational source, including being able to use the cost as a medical expense for tax purposes and being able to use cannabis in recreational locations that would otherwise be banned e.g. in public places. Obtaining MC allows for patients to access formulations tailored to specific conditions and allows access to cannabinoids that are of higher quality.

Overall, this study has shown that physiatrists generally have a positive attitude towards MC. MC has been demonstrated to have positive effects in the management of medical conditions. To increase physiatrists’ comfort using MC to help their patients lead a better life, more research, education and clear guidelines are needed. Even after the legalization for recreational purposes, there are many benefits for patients obtaining MC through medical authorization, such as claiming health coverage or compassionate pricing, writing off cost as a medical expense, using MC in settings where recreational cannabis would be forbidden and receiving guidance from a physician regarding dosing, forms of administration, side effects and balance of THC and CBD. Though many respondents stated they would not feel comfortable using MC after legalization, it would be worth studying how physiatrist’ attitudes and practice patterns have changed. Introducing courses in medical school and training in residency, particularly online learning programs, and small group workshops will be able to increase comfort levels for practitioners.

This study started prior to legalization of recreational cannabis; therefore, respondents answered the survey at a time when their patients did not have legal access to cannabis through recreational means. Although recreational source of cannabis is now available in Canada, medical counselling and authorization remain critical for patients in Physical Medicine and Rehabilitation. It is necessary to ensure that practitioners feel both competent and comfortable at the possibility of considering MC for their patients. Some practitioner attitudes may have changed since legalization, highlighting the need for ongoing research and updated guidance from Health Canada, Canadian Medical Protective Association and provincial regulatory bodies. In addition to this, national-level guidelines with consultations from physiatry specialists would be significantly beneficial. Addressing cannabinoids in medical schools is of utmost importance, not only from the therapeutic side but also from the point of view of counselling patients about safe use and preventing misuse of recreational cannabis. It is evident that teaching in Physical Medicine and Rehabilitation residency programs is lacking, and given our patient population with a high prevalence of pain and spasticity, this needs to be addressed nationally at the Royal College level and within individual programs. With clearer guidelines, more education and better research, physiatrists will be more knowledgeable and to be able to create appropriate treatment plans to improve the lives of their patients.

## References

[1] Andreae MH, Carter GM, Shaparin N, Suslov K, Ellis RJ, Ware MA, et al. Inhaled cannabis for chronic neuropathic pain: a meta-analysis of individual patient data. J Pain 2015; 16: 1221–1232. 10.1016/j.jpain.2015.07.009 26362106 PMC4666747

[2] Modesto-Lowe V, Bojka R, Alvarado C. Cannabis for peripheral neuropathy: the good, the bad, and the unknown. Cleve Clin J Med 2018; 85: 943–949. 10.3949/ccjm.85a.17115 30526755

[3] Lynch ME, Ware MA. Cannabinoids for the treatment of chronic non-cancer pain: an updated systematic review of randomized controlled trials. J Neuroimmune Pharmacol 2015; 10: 293–301. 10.1007/s11481-015-9600-6 25796592

[4] Ware MA, Wang T, Shapiro S, Collet JP. Cannabis for the management of pain: assessment of safety study (COMPASS). J Pain 2015; 16: 1233–1242. 10.1016/j.jpain.2015.07.014 26385201

[5] Nielsen S, Sabioni P, Trigo JM, Ware MA, Betz-Stablein BD, Murnion B, et al. Opioid-sparing effect of cannabinoids: a systematic review and meta-analysis. Neuropsychopharmacology 2017; 42: 1752–1765. 10.1038/npp.2017.51 28327548 PMC5520783

[6] Whiting PF, Wolff RF, Deshpande S, Di Nisio M, Duffy S, Hernandez AV, et al. Cannabinoids for medical use: a systematic review and meta-analysis. JAMA 2015; 313: 2456–2473. 10.1001/jama.2015.6358 26103030

[7] Zajicek J, Fox P, Sanders H, Wright D, Vickery J, Nunn A, et al. Cannabinoids for treatment of spasticity and other symptoms related to multiple sclerosis (CAMS study): multicentre randomised placebo-controlled trial. Lancet 2003; 362: 1517–1526. 10.1016/S0140-6736(03)14738-1 14615106

[8] Pooyania S, Ethans K, Szturm T, Casey A, Perry D. A randomized, double-blinded, crossover pilot study assessing the effect of nabilone on spasticity in persons with spinal cord injury. Arch PMR 2010; 91: 703–707. 10.1016/j.apmr.2009.12.025 20434606

[9] Habib G, Artul S. Medical cannabis for the treatment of fibromyalgia. J Clin Rheumatol 2018; 24: 255–258. 10.1097/RHU.0000000000000702 29461346

[10] Skrabek RQ, Galimova L, Ethans K, Perry D. Nabilone for the treatment of pain in fibromyalgia. J Pain 2008; 9: 164–173. 10.1016/j.jpain.2007.09.002 17974490

[11] Jouanjus E, Raymond V, Lapeyre-Mestre M, Wolff V. What is the current knowledge about the cardiovascular risk for users of cannabis-based products? A systematic review. Curr Atheroscler Rep 2017; 19: 26. 10.1007/s11883-017-0663-0 28432636

[12] Kalla A, Krishnamoorthy PM, Gopalakrishnan A, Figueredo VM. Cannabis use predicts risks of heart failure and cerebrovascular accidents. J Cardiovasc Med 2018; 19: 480–484. 10.2459/JCM.0000000000000681 29879084

[13] Walsh Z, Gonzalez R, Crosby K, Thiessen MS, Carroll C, Bonn-Miller MO. Medical cannabis and mental health: a guided systematic review. Clin Psychol Rev 2017; 51: 15–29. 10.1016/j.cpr.2016.10.002 27816801

[14] Wycoff AM, Metrik J, Trull TJ. Affect and cannabis use in daily life: a review and recommendations for future research. Drug Alcohol Depend 2018; 19: 223–233. 10.1016/j.drugalcdep.2018.07.001 PMC6201696 30149283

[15] Curran HV, Freeman TP, Mokrysz C, Lewis DA, Morgan CJA, Parsons LH. Keep off the grass? Cannabis, cognition and addiction. Nat Rev Neurosci 2016; 17: 293–306. 10.1038/nrn.2016.28 27052382

[16] Hall W. What has research over the past two decades revealed about the adverse health effects of recreational cannabis use? Addiction 2015; 110: 19–35. 10.1111/add.12703 25287883

[17] Ziemianski D, Capler R, Tekanoff R, Lacasse A, Luconi F, Ware MA. Cannabis in medicine: a national educational needs assessment among Canadian physicians career choice, professional education and development. BMC Med Educ 2015; 15: 1–7. 10.1186/s12909-015-0335-0 25888752 PMC4374299

[18] Kondrad E, Reid A. Colorado family physicians’ attitudes toward medical marijuana. J Am Board Fam Med 2013; 26: 52–60. 10.3122/jabfm.2013.01.120089 23288281

[19] Sideris A, Khan F, Boltunova A, Cuff G, Gharibo C, Doan LV. New York physicians’ perspectives and knowledge of the state medical marijuana program. Cannabis Cannabinoid Res 2018; 3: 74–84. 10.1089/can.2017.0046 29662957 PMC5899285

[20] Ebert T, Zolotov Y, Eliav S, Ginzburg O, Shapira I, Magnezi R. Assessment of Israeli physicians’ knowledge, experience and attitudes towards medical cannabis: a pilot study. Isr Med Assoc J 2015; 17: 437–441. 26357721

[21] Karanges EA, Suraev A, Elias N, Manocha R, McGregor IS. Knowledge and attitudes of Australian general practitioners towards medicinal cannabis: a cross-sectional survey. BMJ Open 2018; 8: 1–9. 10.1136/bmjopen-2018-022101 PMC6042562 29970456

[22] Crowley D, Collins C, Delargy I, Laird E, Van Hout MC. Irish general practitioner attitudes toward decriminalisation and medical use of cannabis: results from a national survey. Harm Reduct J 2017; 14: 1–8. 10.1186/s12954-016-0129-7 28086792 PMC5237358

[23] Sharon H, Goldway N, Goor-Aryeh I, Eisenberg E, Brill S. Personal experience and attitudes of pain medicine specialists in Israel regarding the medical use of cannabis for chronic pain. J Pain Res 2018; 11: 1411–1419. 10.2147/JPR.S159852 30104896 PMC6074811

[24] Lucas P. Cannabis as an adjunct to or substitute for opiates in the treatment of chronic pain. J Psychoactive Drugs 2012; 44: 125–133. 10.1080/02791072.2012.684624 22880540

[25] Jimenez XF. Cannabis for chronic pain: not a simple solution. Cleve Clin J Med 2018; 85: 950–952. 10.3949/ccjm.85a.18089 30526750

[26] Ng JY, Gilotra K, Usman S, Chang Y, Busse JW. Attitudes toward medical cannabis among family physicians practicising in Ontario, Canada: a qualitative research study. CMAJ Open 2021; 9: E342–E348. 10.9778/cmajo.20200187 PMC8084545 33849983

[27] Azcarate PM, Zhang AJ, Keyhani S, Steigerwald S, Ishida JH, Cohen BE. Medical reasons for marijuana use, forms of use, and patient perception of physician attitudes among the US population. J Gen Intern Med 2020; 35: 1979–1986. 10.1007/s11606-020-05800-7 32291715 PMC7352011

[28] Walsh Z, Callaway R, Belle-Isle L, Capler R, Kay R, Lucas P, et al. Cannabis for therapeutic purposes: patient characteristics, access, and reasons for use. Int J Drug Policy 2013; 24: 511–516. 10.1016/j.drugpo.2013.08.010 24095000

[29] Boehnke KF, Gangopadhyay S, Clauw DJ, Haffajee RL. Qualifying conditions of medical cannabis license holders in the United States. Health Aff (Millwood) 2019; 38: 295–302. 10.1377/hlthaff.2018.05266 30715980 PMC6398594

